# *ALIXE*: a phase-combination tool for fragment-based molecular replacement

**DOI:** 10.1107/S205979832000056X

**Published:** 2020-02-25

**Authors:** Claudia Millán, Elisabet Jiménez, Antonia Schuster, Kay Diederichs, Isabel Usón

**Affiliations:** a Crystallographic Methods, Institute of Molecular Biology of Barcelona (IBMB–CSIC), Barcelona Science Park, Helix Building, Baldiri Reixac 15, 08028 Barcelona, Spain; b Department of Biology, University of Konstanz, 78457 Konstanz, Germany; c ICREA, Institució Catalana de Recerca i Estudis Avançats, Passeig Lluís Companys 23, 08003 Barcelona, Spain

**Keywords:** phasing, *ARCIMBOLDO*, fragment-based molecular replacement, combination, *ALIXE*

## Abstract

Fragment-based molecular replacement exploits the use of very accurate yet incomplete search models. In the case of the *ARCIMBOLDO* programs, consistent phase sets produced from the placement and refinement of fragments with *Phaser* can be combined in order to increase their signal before proceeding to the step of density modification and autotracing with *SHELXE*. The program *ALIXE* compares multiple phase sets, evaluating mean phase differences to determine their common origin, and subsequently produces sets of combined phases that group consistent solutions. In this work, its use on different scenarios of very partial molecular-replacement solutions and its performance after the development of a much-optimized set of algorithms are described. The program is available both standalone and integrated within the *ARCIMBOLDO* programs. *ALIXE* has been analysed to identify its rate-limiting steps while exploring the best parameterization to improve its performance and make this software efficient enough to work on modest hardware. The algorithm has been parallelized and redesigned to meet the typical landscape of solutions. Analysis of pairwise correlation between the phase sets has also been explored to test whether this would provide additional insight. *ALIXE* can be used to exhaustively analyse all partial solutions produced or to complement those already selected for expansion, and also to reduce the number of redundant solutions, which is particularly relevant to the case of coiled coils, or to combine partial solutions from different programs. In each case parallelization and optimization to provide speedup makes its use amenable to typical hardware found in crystallography. *ARCIMBOLDO_BORGES* and *ARCIMBOLDO_SHREDDER* now call on *ALIXE* by default.

## Abstract 2

The software *ALIXE*, which is available as a standalone program as well as integrated into the *ARCIMBOLDO* programs, combines partial solutions in reciprocal space to increase the signal and reduce the number of intermediate solutions. Its new implementation is faster and can be used by default in the process of phasing with fragments even on modest hardware.

## 1. Introduction   

Extracting information from vast amounts of data with high levels of error and correlation is critical in many sciences. The signal-to-noise ratio (SNR) is a quantitative measure that can be described in terms of the relation between the amount of information present (signal) and the entropy of the system (noise) (Hassan & Anwar, 2010 ▸). Depending on the nature of the noise, it can be reduced by signal averaging (Hassan & Anwar, 2010 ▸). In the field of macromolecular crystallography, recent work has established algorithms and metrics to distinguish, in the case of composite data sets (partial experimental data measured on different crystals), which differences are genuine and which are owing to both systematic and random error (Diederichs, 2017 ▸). The method is general and has been applied successfully to merging crystallographic data sets from microcrystals (Gildea & Winter, 2018 ▸).

The first examples of the relevance of a comprehensive treatment of unclear solutions to enhance the SNR in crystallo­graphic phasing can be found in the low-resolution *ab initio* phasing methods, in which the absence of suitable figures of merit to identify correct solutions was addressed by the combination of consistent solutions (Lunin *et al.*, 1990 ▸, 1995 ▸; Lunin & Woolfson, 1993 ▸; Lunin & Lunina, 1996 ▸). Combination of multiple sources of information is also relevant in the *PanDDA* method (Pearce *et al.*, 2017 ▸), in which structures of a protein in complex with different ligands are used to reduce the noise in maps. One last example relevant to our study is the combination of partial molecular-replacement (MR) solutions (Buehler *et al.*, 2009 ▸).

*ARCIMBOLDO* (Rodríguez *et al.*, 2009 ▸) combines the location of model fragments such as polyalanine helices using *Phaser* (McCoy *et al.*, 2007 ▸) with density modification (Sheldrick, 2002 ▸) and main-chain autotracing (Sheldrick, 2010 ▸; Thorn & Sheldrick, 2013 ▸; Usón & Sheldrick, 2018 ▸) using *SHELXE*. Owing to the difficulties in discriminating correct small substructures, many possible groups of placed fragments have to be tested in parallel. Placement of the fragments with *Phaser* is scored using the log-likelihood gain (LLG), which is the sum of the log likelihoods for individual reflections minus the log likelihoods for an uninformative model. Recent work (Oeffner *et al.*, 2018 ▸; McCoy *et al.*, 2017 ▸) has shown that the signal for an MR search can be estimated before calculation as the expected LLG for a correctly placed model (eLLG). This value will depend on the accuracy of the model, its size and the resolution of the diffraction data. In general, when the LLG score for an initial fragment search is above 60 one can be confident that a correct solution has been found, but such values are seldom reached by placing small fragments. Moreover, the proportion of correct partial structures obtained is frequently extremely low (Schoch *et al.*, 2015 ▸). The main use of our software *ALIXE* (Millán, Sammito, Garcia-Ferrer *et al.*, 2015 ▸) is to combine information from different partial solutions as an effective way to increase the SNR. Combining information may be of applicability for other fragment-based methods, such as *FRAP* (Shrestha & Zhang, 2015 ▸) or *AMPLE* (Bibby *et al.*, 2012 ▸), or may even be of general use in methods combining different sources of phase information to solve challenging structures, such as *Auto-Rickshaw* (Panjikar *et al.*, 2009 ▸, 2017 ▸). As a consequence, the identification of correct solutions may be enhanced, the number of partial solutions to be expanded by density modification and autotracing may be reduced and the starting error in the correct solutions may be lowered. While a proof of concept was attained in the solution of the peptidylarginine deiminase PPAD (Goulas *et al.*, 2015 ▸; Millán, Sammito, Garcia-Ferrer *et al.*, 2015 ▸), the present work describes the current implementation and its use within all *ARCIMBOLDO* programs. Previous algorithms for origin-shift determination have been accelerated, a new FFT-based search has been developed for structures in polar space groups and parallel­ization has been included in the general flow of the program. An optimal parameterization for comparison and merging of the phase sets has been derived. Finally, *ALIXE* has been generalized for use with normal computers within all of our programs or as a standalone program and is illustrated with examples.

## 2. Materials and methods   

### 2.1. Computing setup   

Most structure solutions and tests were run on a local HTCondor version 8.4.5 (Tannenbaum *et al.*, 2002 ▸) grid made up of 160 nodes totalling 225 Gflops. Submitter machines were eight-core workstations with 24 GB RAM running Debian or Ubuntu Linux.

Benchmarking tests and parameterization tests were performed on a workstation with two Intel Xeon Gold 6136 3.0 GHz processors totalling 24 physical cores plus hyperthreading and a DDR4 RDIMM RAM of 8 × 16 GB at 2666 MHz with ECC.

### 2.2. External software   

*Phaser* (McCoy *et al.*, 2007 ▸) is required to perform the MR search of the fragment models. *Phaser* 2.8.*x* versions from the *CCP*4 and *Phenix* distributions were used.

*SHELXE* (Sheldrick, 2010 ▸) is required to provide density modification based on the sphere-of-influence algorithm (Sheldrick, 2002 ▸) and for phase extension and main-chain autotracing (Usón & Sheldrick, 2018 ▸). *SHELXE* 2018 and *SHELXE* 2019 versions were used.

The program *CC_ANALYSIS* (Diederichs, 2017 ▸) reads a (potentially sparse) correlation coefficient (CC) matrix, determines its approximate eigenvalues and eigenvectors to obtain starting values for a set of vectors that each represent a data set, and then refines these vectors to optimally (in a least-squares sense) match their scalar (dot) products to the CC. In this work, the CCs refer to the electron-density map correlations between fragments (see Section 2.3). The *CC_ANALYSIS* executable and documentation are available through XDSwiki (https://strucbio.biologie.uni-konstanz.de/xdswiki/index.php/Main_Page).

### 2.3. Figures of merit and measures of phase similarity   

The figures of merit (FOMs) used in decision making in the fragment location and scoring part of the *ARCIMBOLDO* runs described in this work were the intensity-based LLG and *Z*-score from *Phaser* (Read & McCoy, 2016 ▸) and the correlation coefficient between observed and calculated normalized intensities (CC; Fujinaga & Read, 1987 ▸) calculated by *SHELXE* (Sheldrick, 2002 ▸).

In order to compare phase sets, our tool *CHESCAT* computes two indicators: mean phase differences and map correlation coefficients (equations 1 and 2). The mean phase difference (MPD; equation 1) is an average of all of the phase differences in a set. Its value is zero for identical phase sets and around 90° for uncorrelated phases. The map correlation coefficient (mapCC; equation 2; Lunin & Lunina, 1996 ▸) measures the correlation between two electron-density maps. Its value is 1 for identical phase sets and 0 for uncorrelated phases. The contribution of each reflection to the mapCC and the MPD can be weighted. For the rest of the work presented in this article, the term weighted mean phase error (wMPE) will be used to refer to the error of the phases under study with respect to the true phases, and the term weighted mean phase difference (wMPD) will be used for differences between phase sets in general. Also in this study we will refer to solutions as nonrandom whenever their wMPE is below 80°.
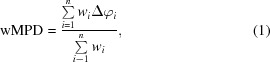


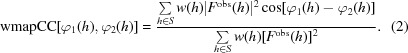



### 2.4. Test data   

Representative cases for phase combination within each of the *ARCIMBOLDO* programs were chosen to cover the various uses of *ALIXE* in polar, nonpolar and *P*1 space groups, where *ALIXE* uses different algorithms.

#### 2.4.1. Hypothetical protein (PDB entry 5vog)   

The crystal structure of a hypothetical protein from *Neisseria gonorrhoeae* with bound ppGpp was downloaded from the PDB (PDB entry 5vog; Seattle Structural Genomics Center for Infectious Disease, unpublished work). The measured data had a resolution of 1.5 Å and the protein crystallized in the nonpolar space group *F*222, with unit-cell parameters *a* = 81.27, *b* = 103.93, *c* = 105.54 Å. The asymmetric unit contains one monomer of 183 residues with 53.69% solvent content. The structure contains 28% helical secondary structure and 32% β-strands.

#### 2.4.2. EIF5   

Crystals of the C-terminal end (residues 232–431) of eukaryotic translation initiation factor 5 (EIF5) belong to space group *P*2_1_2_1_2_1_, with unit-cell parameters *a* = 32.23, *b* = 71.08, *c* = 80.64 Å. The asymmetric unit contains one monomer of 185 residues with 42% solvent content. Data to 1.67 Å resolution were available as amplitudes. The structure (PDB entry 2iu1) was originally solved by experimental phasing (Bieniossek *et al.*, 2006 ▸) and contains 62% helical secondary structure.

#### 2.4.3. Human apo catechol-*O*-methyltransferase   

Crystals of human apo catechol-*O*-methyltransferase (Ehler *et al.*, 2014 ▸) belong to space group *P*1 (PDB entry 4pyi), with unit-cell parameters *a* = 31.596, *b* = 42.638, *c* = 43.663 Å, α = 115.03, β = 95.35, γ = 108.98°. The asymmetric unit contains one monomer of 221 residues with 40% solvent content. Data to 1.35 Å resolution were available as intensities. The structure contains 46% helical secondary structure and 23% β-strands.

#### 2.4.4. GITLR protein   

The crystal structure of the GITRL protein from *Oryctolagus cuniculus* was downloaded from the PDB (PDB entry 4db5; New York Structural Genomics Research Consortium, unpublished work). The crystals belong to space group *F*23, with unit-cell parameters *a* = *b* = *c* = 127.047 Å. The asymmetric unit contains one monomer of 125 residues with 59.53% solvent content. Intensity data are available to a resolution limit of 1.52 Å. The structure comprises 7% α-helices and 49% β-strands.

#### 2.4.5. A46   

The crystal structure of the N-terminal domain of *Vaccinia virus* immunomodulator A46 (PDB entry 5ezu) was solved *ab initio* with *ARCIMBOLDO_BORGES* (Fedosyuk *et al.*, 2016 ▸). The crystals belonged to space group *C*2, with unit-cell parameters *a* = 65.787, *b* = 59.580, *c* = 47.257 Å, α = γ = 90.00, β = 117.70°. The asymmetric unit contains two copies of the monomer with 178 residues and 47.78% solvent content. The data resolution is 1.55 Å and intensity data are available. The entire secondary structure (49%) corresponds to β-strands.

#### 2.4.6. IF1   

Data from the structure of bovine IF1 (PDB entry 1gmj), the regulatory subunit of mitochondrial F-ATPase (Cabezón *et al.*, 2001 ▸), were available to 2.2 Å resolution. The space group was *P*2_1_, with unit-cell parameters *a* = 32.010, *b* = 53.290, *c* = 156.940 Å, α = γ = 90.00, β = 95.89°. The asymmetric unit contains four copies of the monomer with 84 residues and 65% solvent content. All defined secondary structure is α-helical.

#### 2.4.7. Hhed2   

Hhed2 is a halohydrin dehalogenase from a gammaproteobacterium (PDB entry 6qk3). Diffraction data collected at the ALBA synchrotron to 1.6 Å resolution were available. The crystals belong to space group *P*2_1_2_1_2_1_, with unit-cell parameters *a* = 78.02, *b* = 94.86, *c* = 140.27 Å. The asymmetric unit contains four copies of a monomer, totalling 922 residues, with 50% solvent content.

#### 2.4.8. CMI   

The crystal structure of the dimeric immunity protein CMI (PDB entry 4aeq) was originally solved *ab initio* with *ARCIMBOLDO* (Usón *et al.*, 2012 ▸). The space group was *C*222_1_, with unit-cell parameters *a* = 66.06, *b* = 83.55, *c* = 38.28 Å, α = β = γ = 90°. The asymmetric unit contains one monomer with 98 residues and 45% solvent content, which was set to 50% for *SHELXE*. Amplitude data were available to 1.89 Å resolution. The structure comprises 32% helical secondary structure and 27% β-strands.

### 2.5. Distribution of the software   

All of the *ARCIMBOLDO* programs, including *ALIXE*, are distributed through the *CCP*4 suite (Winn *et al.*, 2011 ▸) and are available through the PyPI (Python Package Index) project (https://pypi.org/project/arcimboldo/). The software is provided under the BSD 3-clause licence. The *CHESCAT* executable is also distributed through *CCP*4 and *pip* and is available for download from our website. Documentation and tutorials can be found at http://chango.ibmb.csic.es/.

## 3. Results and discussion   

The present work describes our current implementation and its use within all *ARCIMBOLDO* programs for the following rationale. The target structure is characterized by its set of phases. Any correct partial structure should be consistent with this phase set. *ALIXE* builds an average phase set for each set of consistent solutions. Clustering and origin shift are re-evaluated against each combined phase set until convergence. If all fragments in the structure were included, the process should reconstruct the true phase set. In practice, the sets of correctly placed fragments are incomplete and imperfect, but their correct combination should provide a better approximation than any single solution. An inherent complication is that as the fragment placements are not independent in their generation, sets of wrong consistent probes will also be present. The ability to expand from the combined solutions finally establishes the correctness of the hypothesis.

*ALIXE* is available both as a command-line standalone program and integrated within the *ARCIMBOLDO* programs. In either context, it will operate on a list of files containing phase sets, which in the case of *ARCIMBOLDO* runs will be further characterized by their FOMs (LLG, *Z*-score, CC). A detailed description of the algorithms as well as examples of their application follows.

### 3.1. Implementation   

**Figure 1:**
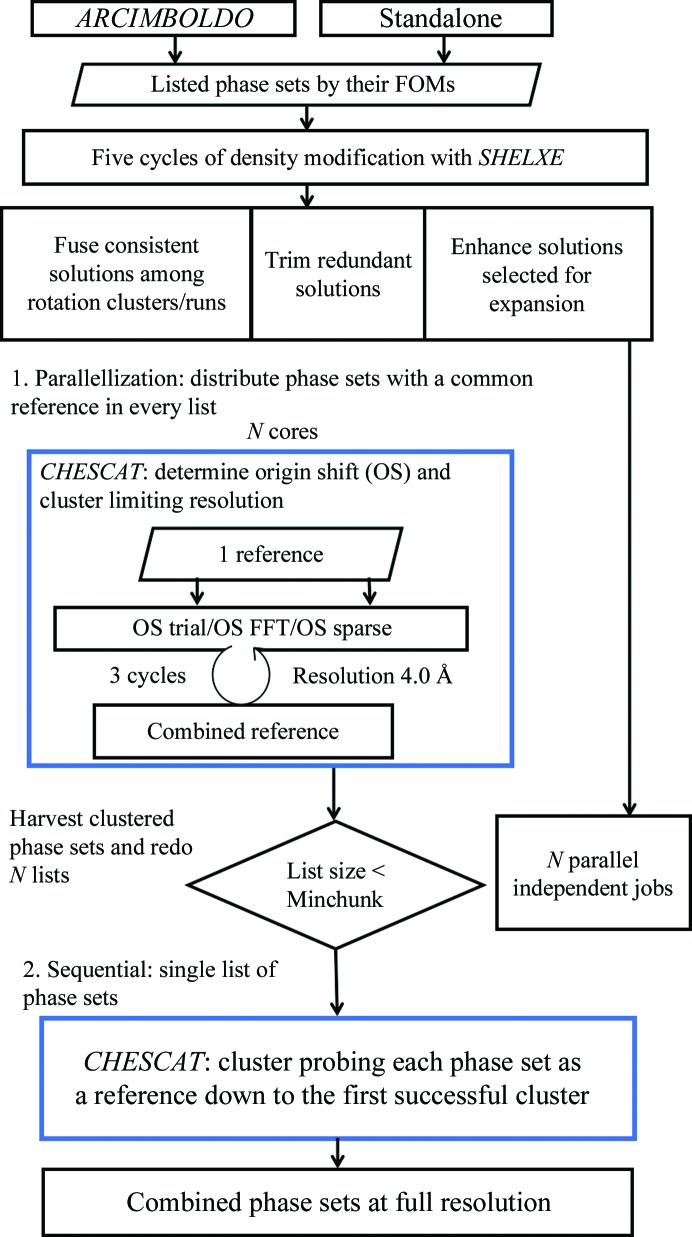
*ALIXE* workflow. The process starts with a set of solutions, to which five cycles of density modification with *SHELXE* are applied. *ALIXE* then splits solutions into groups to be tested in parallel against a common reference, distributing the *CHESCAT* jobs over the specified number of cores. These jobs are performed limiting the resolution. Their output is processed and the solutions that are found to cluster with the reference are removed, and the remaining list is split again over the number of cores. This process is iterated until the list of phase sets is smaller than a threshold size, when *ALIXE* shifts to its sequential version in which *CHESCAT* will probe each phase set as a reference down to the first successful cluster. After all possible references have been tested with both algorithms, the phase sets that were found to cluster are combined at full resolution.

The core algorithm for wMPD-based comparison and merging of multiple phase sets is implemented in a Fortran 95 executable, *CHESCAT*, which is distributed along with the software (see Section 2.5) and which uses a referential clustering procedure. The main program, *ALIXE*, handling the selection of solutions to compare and the clustering strategy is written in Python and is compatible both with Python 2.7.*x* and Python 3.*x* versions. Fig. 1 ▸ shows the overall workflow of *ALIXE*.

The first step involves pre-processing the solutions, typically by running *SHELXE* to compute the phases and sigma weights (Read, 1986 ▸) starting from the positioned fragment. *SHELXE* enhances the discrimination of the correct origin shift through a few cycles of density modification (Millán, Sammito, Garcia-Ferrer *et al.*, 2015 ▸). Alternatively, solutions can be provided in a folder directly in the appropriate map format (.phs) or as coordinate files (.pdb). Within *ARCIMBOLDO* runs, the phase sets are generated during the step that computes the initial CC. The remaining *SHELXE* parameters are taken from the line set in the configuration of the run, which if unset will contain resolution-dependent defaults (Sammito *et al.*, 2015 ▸).

In this work, we have explored strategies to accelerate the clustering process while maintaining its performance. Fragmentation of the comparisons is used both to increase speed through parallelization and to avoid holding an excessive number of solutions in memory. The current core algorithm in *ALIXE* distributes the total number of phase sets to be evaluated over the number of available cores. The subsets of solutions are tested against the same reference, spanning parallel *CHESCAT* processes. Their output is analysed jointly and every phase set that has been found to cluster with the common reference is removed from the input list. Unclustered phase sets will be input to the next round using a fresh reference. This process is repeated until no phase sets remain to be compared. Parallelizing imposes an additional step of phase combination to be performed, joining all of the phase sets that were pulled out by each reference from the different subsets of the input. This provides an opportunity to use different resolution (or varying parameterization in general) for comparison purposes and for merging phases, but it also imposes an overhead compared with a sequential approach. To balance parallel speedup and the aggregation overhead, once the size of the list reaches a given threshold (Minchunk) the algorithm switches to sequential steps in which a single list of phase sets is evaluated.

*CHESCAT* takes a list of phase sets to iteratively build a weighted average from all consistent sets, as determined from the wMPDs. The initial cycle uses the first entry in the list as a reference, and in subsequent cycles differences from the average will be calculated. As the average phases change, more or fewer files join the combined set. The computation of the wMPD involves asserting or inferring the origin shift between the two phase sets under comparison. In the case of nonpolar space groups, where the possible origin is restricted to a few possible positions, all alternative origin shifts for the space group are tested and the one that renders the minimum wMPD is chosen. However, in the case of polar space groups the origin shift will be unconstrained in one or more directions. For such cases, we implement two algorithms that can be used to find the relative shift. In the first algorithm, available since the 2015 implementation, in space groups with a single polar direction the allowed discrete origin shifts are tested and an initial origin shift is estimated in the polar direction using the layer of index 1, which is later refined against all reflections (sparse). In this work, we have also developed and adopted an algorithm using the fast Fourier transform (FFT; Cooley & Tukey, 1965 ▸) to search for the shift in polar space groups. Once the origin shift has been assessed, the files are sorted according to their wMPD to the reference, and if their value is below the threshold set they are merged with a weight proportional to their similarity. A few cycles (by default three) are carried out for convergence. In the first cycle comparisons are performed to a single reference phase set, whereas in subsequent cycles the averaged set becomes the reference.

### 3.2. *ALIXE* for *ARCIMBOLDO*   

**Figure 2:**
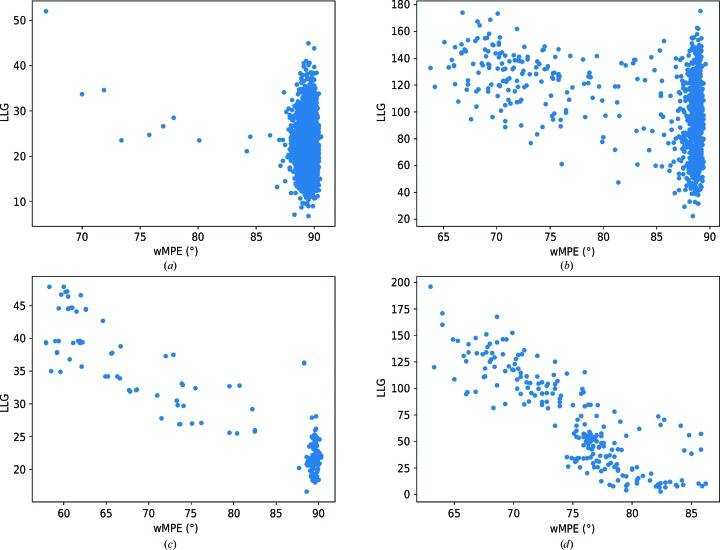
Examples illustrating the typical landscape for partial solutions rendered by phasing with fragments. The points in the scatter plots represent partial solutions from the different *ARCIMBOLDO* test cases. The ordinate shows the LLG score after rigid-body refinement and the abscissa shows the wMPE. (*a*) *ARCIMBOLDO_BORGES* run with the GITRL protein data (PDB entry 4db5) and a library of three antiparallel β-strands. (*b*) *ARCIMBOLDO_SHREDDER* run with the GITRL protein data (PDB entry 4db5) and the template model PDB entry 5l19. (*c*) *ARCIMBOLDO_BORGES* run with the A46 viral structure (PDB entry 5ezu) and a library of three antiparallel β-strands. (*d*) *ARCIMBOLDO_SHREDDER* run with the *P*1 structure (PDB entry 4pyi) and the template model PDB entry 5kva.

Both in its integrated version and as a standalone program, *ALIXE* is typically used to combine solutions from any of the three main *ARCIMBOLDO* programs (Millán, Sammito & Usón, 2015 ▸). Therefore, the landscape of solutions produced is relevant for the design of an efficient clustering procedure. The number of partial solutions as well as the ratio of correct solutions and the indications about their correctness may vary greatly across cases, as illustrated in Fig. 2 ▸, where the characteristics of the phase sets obtained in four different *ARCIMBOLDO_BORGES* (Sammito *et al.*, 2013 ▸) or *ARCIMBOLDO_SHREDDER* runs are plotted. Challenging cases frequently render only a few correct solutions within a large number of total solutions (Fig. 2 ▸ *a*), and the identification of correctness through the LLG may be impossible (Fig. 2 ▸ *b*). Correct solutions tend to show better discrimination in the case of *ARCIMBOLDO_SHREDDER* (Figs. 2 ▸ *c* and 2 ▸ *d*), given the larger fragments and their optimization against the experimental data (Sammito *et al.*, 2014 ▸). If the asymmetric unit contains several copies of the structure, combination of fragments placed on the same and different monomers should be separated into two steps. One important consideration is that the genesis of these solutions is non-independent, and thus consistency is not necessarily evidence of correctness, but it can still be weighted positively and might contribute to discriminating which solutions have improved and setting them apart from the rest.

In the following sections, we will describe the application of *ALIXE* to these cases.

#### 3.2.1. *ALIXE* for *ARCIMBOLDO_SHREDDER* spheres solutions   

*ARCIMBOLDO_SHREDDER* in its spheres mode (Millán *et al.*, 2018 ▸) produces a set of compact, overlapping models starting from a distant homologue template. In the course of the run, a large number of possible partial solutions are produced, corresponding to best-scored locations of different models clustered around differentiated rotation angles. Such models are modified relying on the experimental data in alternative ways, such as decomposition and refinement with *gyre* and *gimble* (McCoy *et al.*, 2018 ▸), normal-mode deformation (McCoy *et al.*, 2013 ▸) or pruning to optimize the CC (Sheldrick & Gould, 1995 ▸) or LLG (Oeffner *et al.*, 2018 ▸). The overlapping nature of the models, which all represent parts of a general hypothesis for the target fold, eases the reconstitution of a more complete solution and provides a favourable context for improvement. On the other hand, given that the process modifies the model fragments with internal degrees of freedom, superposition in real space would entail an artificial decision about the core to match. Agreement in reciprocal space provides a more natural metric. Phase combination with *ALIXE* is frequently essential to improve the starting phase set prior to density modification and autotracing and is a key contribution to the *ARCIMBOLDO_SHREDDER* method, as shown by the solution of a number of previously unknown structures.

*Combining overlapping solutions*. In the present work, the algorithms in *ALIXE* and the wMPD thresholds used were revisited in cases ranging from cubic, as in the *F*23 structure of the GITRL protein (PDB entry 4db5), to *P*1, as exemplified in the test structure PDB entry 4pyi. For the cubic structure, an *ARCIMBOLDO_SHREDDER* run with the template model PDB entry 5l19, which shares barely 17% sequence identity, produced 65 nonrandom solutions from a total of 171. The best solution shows a wMPE of 57.9°. *ALIXE* rendered three correct phase clusters, the best of which significantly improved the wMPEs to the final structure to 48.7° and 52.10°, respectively. In the case of the triclinic test structure PDB entry 4pyi, *ARCIMBOLDO_SHREDDER* spheres produced 447 partial solutions, of which 189 were nonrandom and distributed along a rather continuous wMPE landscape (shown in Fig. 1 ▸ *d*). The best wMPE from a single solution was 63.0°. The clustering process forms 89 clusters, including three correct clusters, with the best of them having a wMPE of 65.9°. Thus, no real improvement is observed in this case, but also no significant deterioration despite the unconstrained origin shift. Also, the present *ALIXE* implementation allows these solutions to be clustered, whereas the task was hopelessly time-consuming in the previous version.

Our tests on the threshold to combine solutions for overlapping fragments have confirmed the value of 60° as a good compromise to recognize consistent solutions.

*Exploring relations between solutions using *CC_ANALYSIS**. A mode has been included in *ALIXE* that prepares the input required to perform an analysis using the map correlation coefficients between possible solutions and the program *CC_ANALYSIS* (Diederichs, 2017 ▸) described in Section 2.2. *CC_ANALYSIS* requires as input all of the pairwise map correlation coefficients between phase sets. The algorithm implemented in *ALIXE* to compute these comparisons is embarrassingly parallel, as they are totally independent computations.

**Figure 3:**
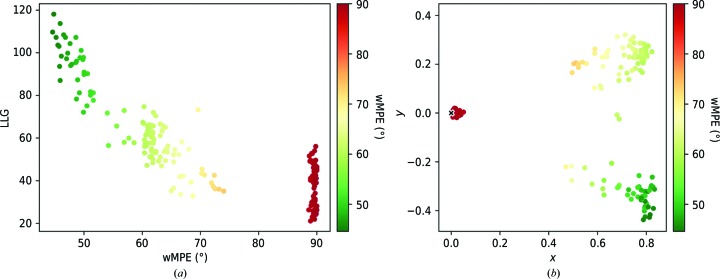
*CC_ANALYSIS* tests on the *F*222 hypothetical protein (PDB entry 5vog) solutions from an *ARCIMBOLDO_SHREDDER* run using PDB entry 5bqp as a template model. (*a*) Plot characterizing the solutions in terms of figures of merit, with the wMPE as the abscissa and the LLG as the ordinate. A clear discrimination is observed. (*b*) The origin is marked by a black cross. The axes are unitless. The correctness of solutions is described by a colour gradient according to their wMPE, with green indicating a lower wMPE. The two correct differentiated populations separated solutions refined by *gyre* in *Phaser* (dark green, more correct) from those lacking internal degrees of freedom (lighter green).

To illustrate the use of this mode, the test case PDB entry 5vog was chosen. Solutions are from an *ARCIMBOLDO_SHREDDER* run using chain *C* from PDB entry 5bqp as a template model, which has 28% sequence identity and an r.m.s.d. of 1.0 Å to the target. The data are described in Section 2.4.1. Fig. 3 ▸(*a*) characterizes the solutions from the run. Fig. 3 ▸(*b*) shows the results from the output obtained using *CC_ANALYSIS*. The separation of correct and incorrect solutions is clear-cut and the correct solutions were assigned vectors of lengths of up to 0.9, indicating a high SNR. The incorrect solutions clustered around the origin. The correct solutions were further separated into two clusters. Upon closer examination, it became clear that the solutions in one cluster had been refined against the rotation function by *gyre* refinement in *Phaser* and therefore had a lower wMPE. This is an interesting example of how *CC_ANALYSIS* can distinguish between two populations that have systematic differences.

*Combining solutions from different monomers in the asymmetric unit*. In the general case, if the asymmetric unit is expected to contain only one monomer, a single step of phase combination will be performed for each rotation cluster. However, if multiple copies are expected, two phase-clustering steps will be performed. In the first step, phase combination is performed within rotation clusters and the resulting combined phase sets are then used for a second round of combination using a higher tolerance (87°) on all of the available clusters from the first round. A successful example of the application of this strategy has recently been published (Millán *et al.*, 2018 ▸), in which the previously unknown structure of Hhed2 was determined. The phase set that led, upon density modification and autotracing, to a full solution of the structure was obtained by merging, using *ALIXE*, partial solutions from three different monomers found in two different rotation clusters from the *ARCIMBOLDO_SHREDDER* run.

#### 3.2.2. *ALIXE* for *ARCIMBOLDO_BORGES* solutions   

In *ARCIMBOLDO_BORGES* (Sammito *et al.*, 2013 ▸) a library of superimposed models is used to represent a given geometry. Such libraries are generated with our software *ALEPH* (Medina *et al.*, 2020 ▸) and contain thousands of variations of a given small local fold, such as for example three antiparallel β-strands or two parallel α-helices. Even if such models constitute a piece of the tertiary structure, they are general and unspecific, so they may fit a given structure in multiple ways simultaneously, including in an overlapping manner. Therefore, reconstituting their overlap in reciprocal space might complete the starting hypothesis while adjusting for geometrical deviations, whereas in real space it would be necessary to advance a supposition about the final structure in order to decide on how to best combine them. The test case PDB entry 5ezu (Section 2.4.5) was selected to exemplify the use of *ALIXE* in *ARCIMBOLDO_BORGES* runs. It is an all-β structure in which β-strands are aligned in the crystal, forming extended β-sheets. The structure was solved using our CCP4-distributed library, representing a set of three antiparallel β-strands (named strands udu in *CCP*4*i* ). The run produces 905 solutions distributed among four rotation clusters. Nonrandom solutions are present in all four clusters: 152 in total, with the best having a wMPE of 63.8°. Their relative figures of merit (LLG and wMPE) are shown in Fig. 2 ▸(*c*). Ten nonrandom clusters are formed by *ALIXE*; the best, with a wMPE of 60.4°, joins 26 solutions. Most nonrandom clusters, after density modification and autotracing, reach a CC of over 30% and allow successful model building of the complete structure.

#### 3.2.3. *ALIXE* using solutions from *ARCIMBOLDO_LITE*   

In the case of the standard *ARCIMBOLDO_LITE*, in which a sequential search of fragments builds up a solution, *ALIXE* is not used by default after a single-copy search because even if phase combination were to succeed, the increase in the signal upon the correct placement of a second fragment is very high, enhancing the identification of correct solutions and providing a superior strategy (Oeffner *et al.*, 2018 ▸). Furthermore, the search for single models in the case of helices is computationally not very demanding. Still, some ways are provided to perform clustering on partial solutions from *ARCIMBOLDO_LITE*. In its coiled-coil mode, in which thousands of correlated solutions are often found and discrimination of the correct solutions may not be possible before extension, *ALIXE* can be used to reduce the number of redundant hypotheses and to provide a better starting map for autotracing.

*Standard *ARCIMBOLDO_LITE**. *ARCIMBOLDO_LITE* (Sammito *et al.*, 2015 ▸) uses single models and is the *ARCIMBOLDO* program that is most commonly used on limited hardware such as a single workstation or a laptop. Therefore, using as references those solutions that are going to be sent to expansion and autotracing with *SHELXE* anyway, it was attempted to find other compatible solutions with the test case PDB entry 2iu1 (Section 2.4.2). This structure can be solved with the latest version of *ARCIMBOLDO_LITE*, searching for two helices of ten residues. The runs were performed on a machine with four cores. The main Python process in *ARCIMBOLDO_LITE* will be running on one of the cores and the other three will be used to distribute the external executables jobs. By default, only five solutions will be trialled for density modification and autotracing. The run searching for helices of ten residues produces five nonrandom solutions at the second fragment search, with the best wMPE for a single solution being 65.9°. The five solutions that are sent to expansion generate two different clusters when used as references, one characterized by a wMPE of 64.2° to the final structure (six solutions merged) and another by a wMPE of 62.4° (eight solutions merged).

Thus, in general cases clustering can be used within *ARCIMBOLDO_LITE* to enhance the starting solutions and to diversify the hypotheses to be trialled.

**ALIXE* using solutions from the coiled-coil mode in *ARCIMBOLDO_LITE**. The performance of *ALIXE* as a tool to reduce the redundancy of solutions in the *ARCIMBOLDO_LITE* coiled-coil mode (Caballero *et al.*, 2018 ▸) was explored with a set of test structures proposed by the group developing the phasing software *AMPLE* (Thomas *et al.*, 2015 ▸), and it will be exemplified with the test case of bovine IF1 (PDB entry 1gmj). This structure can be solved in *ARCIMBOLDO_LITE* searching for four ideal polyalanine α-helices of 25 residues and activating the coiled-coil mode defaults. The helices in the deposited structure range between 53 and 65 residues. For each of the helix searches (from fragment 1 to 4), all solutions were considered for the purpose of the experiment, whereas usually a hard limit is set on the total number of solutions allowed for each step in *ARCIMBOLDO* to prevent collapsing the hardware. Also, instead of performing the clustering within rotation clusters from *ARCIMBOLDO*, all of the solutions were compared together. Two tolerances were tried: 60°, which is our default threshold for merging overlapping solutions from *ARCIMBOLDO_BORGES* and *ARCIMBOLDO_SHREDDER*, and 30°, which aimed to identify very similar solutions and to avoid merging too much noise and deteriorating the solutions.

**Figure 4:**
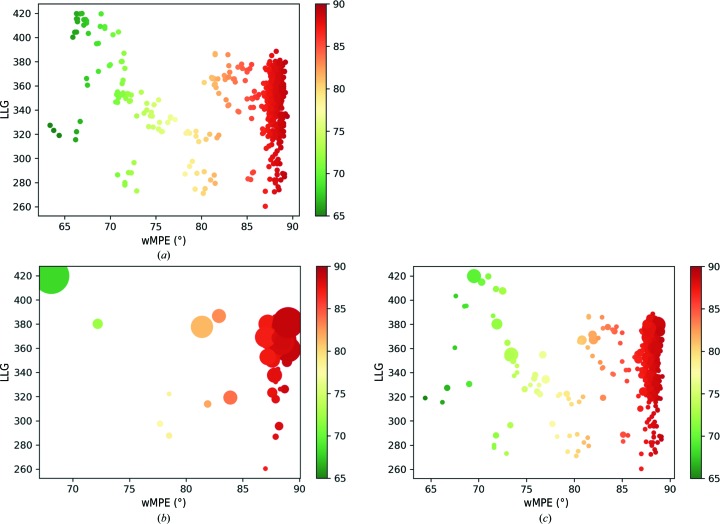
Analysis of the clusters rendered by *ALIXE* after the search for a second fragment in the coiled-coil mode of *ARCIMBOLDO_LITE* for the IF1 protein structure (PDB entry 1gmj). The dots in the scatter plots represent either single solutions or combined clusters. The size of the circles is proportional to the number of single solutions joined in that cluster. The colour and the value in the abscissa represent the wMPE, and the ordinate represents the LLG value for the top solution in the cluster. (*a*) Original single solutions. (*b*) Clusters under a 60° wMPD tolerance. (*c*) Clusters under a 30° wMPD tolerance. It can be observed that a 30° tolerance results in a more differentiated landscape in which the number of solutions is reduced while keeping sufficient diversity to avoid deterioration in the clusters with the best wMPEs.

After the first fragment placement, only two out of 50 solutions were correct, each of which was part of a different rotation cluster of the two found at this stage. In the second fragment search, 102 out of 629 solutions were correct, distributed among seven rotation clusters. In the third and fourth fragment searches the number of correct solutions was much higher, so we performed our analysis at the stage of the second helix placement. Fig. 4 ▸ displays the results of the phase clustering and their comparison with the single solutions. At a tolerance of 30° wMPD, the 629 solutions (Fig. 4 ▸ *a*) were reduced to 324 clusters (Fig. 4 ▸ *b*), whereas at 60° tolerance only 47 solutions remained (Fig. 4 ▸ *c*). However, in the second case the higher error relative to the real structure suggests that the inclusion of noise is deleterious. At 30°, while the error is similar, clustering provides a means of choosing the references to each cluster as the solutions to pursue in the search for the next fragment. This halves the amount of fixed solutions at the start of the next step.

#### 3.2.4. Combining solutions from model helices in *ARCIMBOLDO_LITE* with libraries of β-sheets in *ARCIMBOLDO_BORGES*   

Fragments of different structural nature, such as α-helices and β-sheets, will necessarily produce solutions matching different parts of the structure, and therefore their combination will bring together complementary information. This also implies that their relative wMPDs will be high, close to the 90° presented by unrelated phase sets. To test the applicability of the combination of such fragments, *ARCIMBOLDO_LITE* searching for an helix of 14 residues and *ARCIMBOLDO_BORGES* using a library of three antiparallel β-strands were run on the test structure PDB entry 4aeq (Section 2.4.8). It has to be mentioned that either run would by itself solve this small structure, in which the models represent a fair fraction of the scattering. However, our interest was to explore in the best possible scenario the phase differences that could be expected when combining such independent types of fragments and to derive a strategy for subsequent use. The *ARCIMBOLDO_LITE* run produces seven solutions, of which four are correct (wMPE between 57.4° and 63.9°) and three are incorrect (wMPE between 87.8° and 88.9°). The four correct solutions indeed match overlapping sections of the 28-residue helix in the final structure. The *ARCIMBOLDO_BORGES* run produces 3242 solutions, of which only 11 are correct, with an wMPE ranging from 66.7° to 76.8°. Correct solutions are present in three of the four rotation clusters. Our first analysis involved the comparison of the correct solutions only. Using a tolerance threshold of 60° in wMPD, the four correct *ARCIMBOLDO_LITE* solutions cluster together in *ALIXE* forming a cluster with a wMPE of 64°, and the 11 correct solutions from *ARCIMBOLDO_BORGES* do the same, forming one cluster with a wMPE of 67°. If this threshold is set to 80° in wMPD, the 15 correct solutions from both runs are merged together and form a cluster with a wMPE of 58.50°. The following step tested the case in which the presence of incorrect solutions in the pool may introduce noise. For this experiment, we used the solutions from each of the four *ARCIMBOLDO_BORGES* rotation clusters and from the helices and trialled different wMPD thresholds. Using a threshold of 83°, correct clusters blending solutions from α-helices and β-sheets were formed. The best cluster was achieved in the comparison between the solutions from one of the *ARCIMBOLDO_BORGES* rotation clusters, in which nine phase sets were merged, producing a map with a wMPE of 56.60°. This cluster combines the three correct solutions from β-sheets along with two incorrect solutions and the four correct solutions from the helices.

### 3.3. Accelerating performance: timing benchmarks   

The performance of the algorithms described is heavily dependent on the amount of input, derived from the (usually large) number of phase files under comparison but also from their size in terms of number of reflections and symmetry order, and therefore the parallelization and optimization strategies implemented have been tested on different input sets of solutions to evaluate the timings, to identify bottlenecks and to benchmark the speedup.

The current implementation of *ALIXE* is described in Section 3.1. In the previous version of *ALIXE*, *CHESCAT* was called sequentially to perform clustering over a list of solutions sorted according to phasing figures of merit and comprising phase sets to a maximum resolution of 2.0 Å. Referential clustering was performed in which the top solution was used as an initial reference to identify other phase sets in the list below a given wMPD threshold from the combined set. All phase sets in this cluster were combined and removed from the list. This process was repeated until the list was finished. Every clustering attempt was performed sequentially and involved calling on *CHESCAT* from *ALIXE* and interpreting its output.

#### 3.3.1. Limiting the resolution for phase-set comparison   

One potential drawback of using a parallel algorithm was turned into an advantage: aggregation requires a second merging step on clusters that have been formed using a common reference. This allows different parameterization during comparison than for the final formation of the clusters to be expanded. Our previous resolution default for phase comparison was set to 2.0 Å. In this work, a set of experiments using resolution cutoffs to 2.0, 2.5, 3.0, 3.5 and 4.0 Å were performed. The test cases comprised all partial solutions from two *ARCIMBOLDO_LITE*, two *ARCIMBOLDO_SHREDDER* and two *ARCIMBOLDO_BORGES* runs.

In all of our test cases, limiting the resolution even to 4.0 Å does not compromise the formation of correct clusters, as they eventually reach the same wMPE. This indicates that limiting the resolution to 4.0 Å to select consistent phase sets does not impair the procedure, although the number of total clusters is reduced in every case at lower resolution. The differences are found in the treatment of incorrect or borderline phase sets, so the high-resolution reclustering renders the same final best sets. This implies that not all data are required to identify the origin shift, so we can establish 4.0 Å as the default resolution to compare phase sets. The *ARCIMBOLDO_SHREDDER* *P*1 test-case solutions constituted the only exception, where equivalent but not identical phase sets were formed. In the *P*1, unconstrained case, determination of the common origin is more error-prone and therefore a more conservative cutoff of 3.5 Å will be adopted in this space group. Moreover, the time consumed is reduced in all runs, such as the case shown in Table 1 ▸, PDB entry 5ezu, in which the reduction in time reaches 11 min.

#### 3.3.2. Using a sparse or an FFT algorithm for the origin-shift search   

The performance of the FFT algorithm for the origin shift was assessed in three test structures, one in space group *P*1 (PDB entry 4pyi; 38 358 reflections), one in space group *C*2 (PDB entry 5ezu; 23 425 reflections) and one in space group *P*6_3_ (PDB entry 5ohu; 51 143 reflections). In all three cases the clustering results were equivalent and the correct clusters were formed. As shown in Table 1 ▸, in space group *P*1 the FFT algorithm was 126 times faster (from 443 to 4 min) than the approximation used in the sparse algorithm. In the case of PDB entry 5ezu, the timing was not significantly different with either algorithm. Only in the case of the large structure PDB entry 5ohu was the FFT algorithm slightly slower (by 3 min) than the sparse algorithm. As the FFT algorithm maintained the same overall results as the sparse algorithm, but substantially reduced the time in *P*1, it will be implemented as the default parameterization for this particular space group. For other polar space groups, the choice will depend on the number of reflections.

#### 3.3.3. Testing the maximum speedup and optimal number of cores for the parallelization   

**Figure 5:**
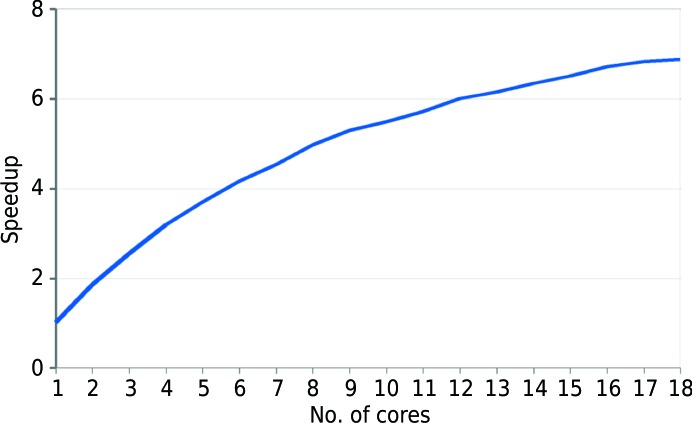
The ordinate shows the speedup using the parallel algorithm and the abscissa the number of cores used. The speedup represents the ratio of the runtime of the sequential algorithm to the runtime of the parallel algorithm to solve the same problem using a given number of processors.

In order to better manage the hardware resources available to *ALIXE* and to characterize the efficiency of our parallelization, Amdahl’s law was used to compute the speedup derived from increasing the number of cores. The efficiency of the algorithm was estimated to be 91%. As can be seen from Fig. 5 ▸, the speedup effectively increases up to ten cores and then reaches a plateau. In fact, it can be observed that the decrease in time is negligible between ten and 18 cores. The impact of the parallelization using ten cores is illustrated in Table 1 ▸, where in the case of the cubic structure PDB entry 4db5 the time is reduced from 1402 to 257 min. In view of these results, the defaults in *ALIXE* will be set to use the number of physical cores minus 1, with a maximum of ten cores in the case of systems with more than 12 CPUs.

#### 3.3.4. Optimizing the sequential part of the algorithm   

**Figure 6:**
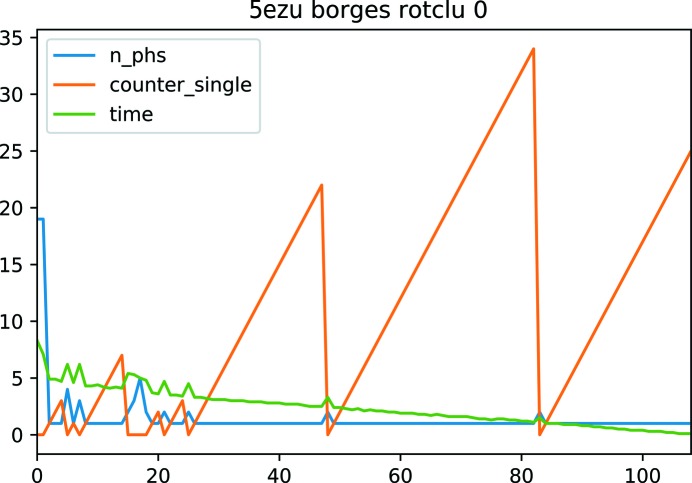
On the abscissa, the index of the order in the sorted list of input phase sets for use as references is shown. The ordinate shows values for the three magnitudes represented in the plot: n_phs is the number of phase sets joined in each clustering attempt, time is the time spent and counter_single is a counter that is reset to zero each time a clustering attempt succeeds and increases when a reference does not cluster with anything.

As discussed in the previous section, there are limits to the speedup that one can achieve by splitting the tasks, as there is an overhead in their aggregation. Furthermore, from the point at which no more clustering events are successful, the aggregation overhead is going to be wasted. An experiment was performed to analyse the frequency at which a new cluster was being formed while the list of initial solutions was being trialled. The results for the test case PDB entry 5ezu are shown in Fig. 6 ▸. As can be seen, while the list is traversed the clustering success decreases, with most clusters (whether correct or not) being formed at the beginning. The results suggest that swapping to the sequential algorithm when cluster formation is going to be more unlikely is a convenient strategy. The results also prompted the development of an improvement for the sequential part of the clustering, involving the reduction of the number of inter-calls between *ALIXE* and *CHESCAT* when processing the list of phase sets. We have included an instruction in *CHESCAT* that uses the first phase set of the list given by *ALIXE* as a reference and, instead of finishing if no cluster is formed, continues the process using the next phase set in the list as a reference until it succeeds in forming a cluster or the list is finished. The results for two of the largest test cases (*ARCIMBOLDO_BORGES* runs from PDB entries 5ezu and 4db5) showed that this simple change made the runs up to nine times faster while preserving equivalent clustering results. In Table 1 ▸, the results for PDB entry 4db5 are shown, where the time is reduced from 1402 to 149 min.

#### 3.3.5. Overall speedup   

The time consumed in phase combination will depend on the structure, the number of solutions and the hardware. Previous timings referred to the effect of isolated modifications. To give a general idea of the performance reached in the new implementation, the 162 phase sets from a single rotation cluster in the PDB entry 5ezu *ARCIMBOLDO_BORGES* run were clustered on a laptop with four physical cores. Whereas a single-core run under previous conditions would take 6.35 min, our present implementation using all defaults runs for 1.40 min on a single core and 1.28 min distributed on three cores. This shows that the overhead of clustering is negligible on the *ARCIMBOLDO* time scale, and its use is now set as default.

## 4. Concluding remarks   

Combining phase sets derived from partial solutions in fragment phasing increases their information content and is therefore effective in providing a better start for the extension into a full structure through density modification and autotracing.

*ALIXE*, which provides a framework for the comparison and merging of multiple partial solutions, has been extended, its parameterization optimized and its speed increased to allow general use of its algorithms within the *ARCIMBOLDO* programs or independently. Within *ARCIMBOLDO*, a choice can be made between the exhaustive clustering of all solutions or limited combination to enhance those solutions already selected for expansion. Avoiding redundancy is also of interest, especially on limited hardware or when possible solutions are produced in large numbers.

The present implementation incorporates parallelization in balance with a sequential stage. Data resolution is limited to 4.0 Å during the stage where clusters are built and origin shifts are determined in the *CHESCAT* executable. During the sequential stage, inter-calls between *ALIXE* and *CHESCAT* have been minimized. The wMPD provides the metric for clustering, but the map correlation coefficient is also calculated by default and may be used for *CC_ANALYSIS*. We have increased the usability of the software even with limited hardware and derived an optimal parameterization. Finally, as a result of this work, *ARCIMBOLDO* now uses *ALIXE* by default. *ALIXE* is also distributed as a standalone program.

## Figures and Tables

**Table 1:** Illustrative examples of timings with different parameterizations and algorithms

PDB code	Space group	No. of solutions (No. correct)	Best wMPE single (°)	Algorithm	*d* (Å)	No. of clusters (No. correct)	Best wMPE (°)	Time (min)
4pyi	*P*1	224 (189)	63.0	FFT	2.0	9 (6)	65.5	4
				Sparse	2.0	9 (6)	65.5	443
5ezu	*C*2	905 (152)	63.8	FFT	2.0	73 (10)	60.4	30
					2.5	60 (9)	60.4	26
					3.0	50 (8)	60.4	23
					3.5	45 (8)	60.4	21
					4.0	42 (8)	60.4	19
4db5	*F*23	3748 (7)	66.9	Parallel 1 core	2.0	48 (2)	61.8	1402
				Parallel 10 cores	2.0	48 (2)	61.8	257
				Seed0	2.0	48 (2)	61.8	1402
				Seed1	2.0	45 (2)	61.8	149
